# The Role of Immune Regulatory Cells in Nontraumatic Osteonecrosis of the Femoral Head: A Retrospective Clinical Study

**DOI:** 10.1155/2019/1302015

**Published:** 2019-11-20

**Authors:** Jinhui Ma, Juncheng Ge, Fuqiang Gao, Bailiang Wang, Debo Yue, Wei Sun, Weiguo Wang

**Affiliations:** ^1^Department of Orthopaedic Surgery, Center for Osteonecrosis and Joint Preserving & Reconstruction, China-Japan Friendship Hospital, Beijing 100029, China; ^2^Department of Orthopaedic Surgery, Peking University China-Japan Friendship School of Clinical Medicine, Beijing 100029, China

## Abstract

The immunologic factors have been implicated in the pathogenesis of osteonecrosis. We aimed to investigate the potential role of immune regulatory cells in the development of osteonecrosis of femoral head (ONFH). Sixty-seven patients diagnosed with ONFH and fifty-eight age-, height-, and weight-matched healthy subjects were included in this retrospective study between September 2015 and September 2018. The flow cytometry was used to test the count, percentage, and ratio of T and B lymphocyte subsets in peripheral blood. The T and B lymphocyte levels were compared among different ARCO stages, CJFH types, and etiology groups. The total lymphocyte count, CD3^+^T cells, Ts cells (CD3^+^CD8^+^), B-1 cell count, and B-1 cells (CD5^+^CD19^+^) were significantly higher in the patients with ONFH than those in the control subjects. The percentage of T lymphocytes in the patients with ARCO IV stage was significantly smaller than that in the ONFH patients with ARCO II and III stages. The percentage of inhibitory T lymphocytes in patients with CJFH type L3 was significantly smaller than that in the patients with types L1 and L2. In terms of the different ONFH etiologies, the total lymphocyte count and Ts cells (CD3^+^CD8^+^) were significantly lower in the ONFH patients induced by excessive alcohol intake than those in the idiopathic ONFH patients. Our results seem to indicate that immune regulatory cells, such as T and B lymphocytes, play an important role in the pathogenesis of ONFH. The development and progression of ONFH may be associated with immune system imbalance.

## 1. Introduction

Nontraumatic osteonecrosis of the femoral head (ONFH) is a refractory and progressive disease that frequently leads to femoral head collapse and degenerative changes to the hip joint [1]. It can be entirely asymptomatic, detected only by imaging, or severe, requiring surgical intervention, which brings disaster for patients and families [2]. Nontraumatic ONFH has been often related to corticosteroid usage, chronic alcohol consumption, infection, hyperbaric events, storage disorders, marrow infiltrating diseases, coagulation defects, immoderately low or high temperatures, and some autoimmune diseases [3, 4].

Although there are many risk factors for osteonecrosis of the femoral head, the specific pathogenesis of osteonecrosis is not clear. Currently, widely accepted theories include: blood transportation obstacle [5, 6], lipid metabolism disorder [7], intraosseous hypertension [8], bone cell apoptosis [9], immune factors [2]. During the development of avascular necrosis of femoral head, bone absorption is greater than bone formation, which is an important factor leading to femoral head collapse. Meanwhile, some scholars have found that immune imbalance plays an important role in the pathological process of femoral head necrosis [10, 11]. Under normal circumstances, the function of osteoblasts and osteoclasts maintains a balance, and immune cells play an important role in maintaining this balance. After femoral head necrosis occurs, the immune balance is broken due to the large amount of chemokines and cytokines released by local necrotic cells. If this imbalance is difficult to correct and osteoclasts gradually become more active than osteoblasts, then bone destruction will be difficult to repair and eventually lead to irreversible collapse of the femoral head. At this stage, joint replacement may be the only solution for patients. In addition, patients with autoimmune diseases have a higher probability of femoral head necrosis than normal persons, which may be related to long-term use of glucocorticoids, vasculitis, and immune dysfunction [11].

In ONFH, osteoblasts and osteoclasts play an important role in bone tissue reconstruction. The close relationship between immune cells and bone cells can be demonstrated by direct or indirect action on bone differentiation through the receptor activator κB factor ligand (RANKL)/the receptor activator κB factor (RANK)/osteoprotegerin (OPG) system. Stimulation or inhibition is related to immunocyte subsets, cytokines, and local factors. Besides, some scholars believe that osteoclasts and immune cells have a common origin [12, 13]. The combination of RANKL expressed by osteoblasts and RANK expressed by osteoclast precursors can promote the differentiation, activation, and maturation of osteoclasts. In addition, osteoblasts secrete OPG, which is specifically combined with RANK to inhibit osteoclast differentiation and promote osteoclast apoptosis. The balance between RANKL and OPG determines the rate of bone resorption. The stimulation and inhibition of T cells on osteoblasts and osteoclasts are closely related to T cell subsets and cytokines. Activated T cells can produce RANKL and OPG, and early research suggests that Th1 cells are the main source of RANKL. Meanwhile, the study also found that CD4^+^ Th17 cells can produce higher activity and a high level of RANKL, express cytokines TNF-2, IL-23, and IL-17, and promote RANKL expression [14]. Regulatory T cells, a subset of inhibitory T cells, can bind to B7-1 and B7-2 in osteoclast precursors via cytotoxic T lymphocyte associated protein (CTLA)-4, and the combination inhibits osteoclast damage to the bone [15]. B cells also produce RANKL and secrete OPG, and studies have shown that mice lacking bone marrow B cells often develop osteoporosis because they lack the OPG secreted by B cells. This osteoporosis can be corrected by receiving adoptive B cells [16].

Osteoimmunology is a relatively new branch of immunology. At present, there are not many researches conducted in this branch. In our previous studies, we found that IL-33 was associated with the prognosis of patients with ONFH [17]. This gives us the inspiration to explore the role of immune cells and immunomodulatory cells in ONFH. Therefore, we use a similar methodology to explore this issue. In this study, we collected peripheral blood from patients with ONFH and patients in the non-ONFH control group to investigate the potential role of immune regulatory cells in the development of ONFH. We expect to provide a preliminary guidance for the further study of osteoimmunology in ONFH and, if possible, to explore the treatment of ONFH from the perspective of immunology.

## 2. Materials and Methods

### 2.1. Study Population

This was a retrospective clinical control study. Sixty-seven patients were diagnosed with nontraumatic femoral head necrosis (ONFH) between September 2015 and September 2018. These patients were diagnosed by more than two professional orthopedic surgeons in our department, and the diagnosis was combined with clinical history, physical examination, X-ray, and MRI imaging evaluation. The inclusion criteria were the diagnosis of nontraumatic ONFH. Patients who met the following criteria were excluded: history of trauma, history of active hip infection, inflammatory disease, cardiovascular disease, immune deficiency, HIV infection, diabetes, kidney disease, or previous surgery on the hip with ONFH. The control group included fifty-eight healthy subjects admitted to the hospital at the same time. Age, gender, height, and weight in the control group were matched with the ONFH group. This study was approved by the China-Japan Friendship Hospital and was carried out in accordance with the Helsinki declaration. Written informed consent was obtained from all subjects or their guardians [17].

**Figure 1:**
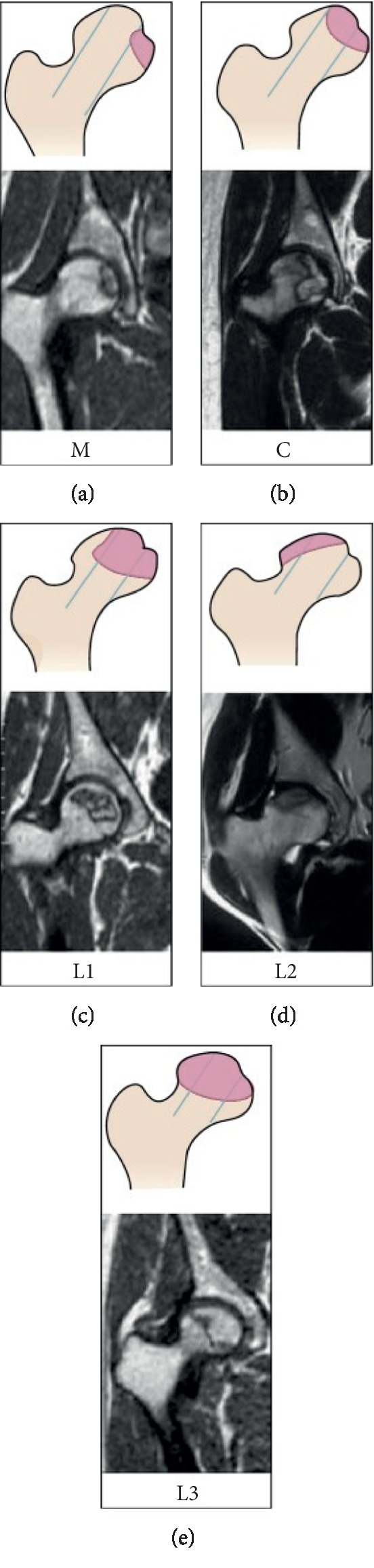
Schematic diagram and magnetic resonance image of China-Japan Friendship Hospital (CJFH) classification for osteonecrosis of the femoral head based on three pillars [19]. Type M necrosis involves the medial pillar. Type C necrosis involves the medial and central pillars. Type L1: necrosis involves the three pillars but the partial lateral pillar was preserved. Type L2: necrosis involves the entire lateral pillar and part of the central pillar. Type L3: necrosis involves the three pillars including the cortical bone and marrow.

Combined with clinical symptoms and imaging findings, patients in the ONFH group were evaluated according to Harris hip score (HHS) [18], CJFH type [19] (Figure 1), and ARCO classification system [20]. The characteristics of the included ONFH patients are presented in Table 1. The mean patients' age was 43.6 ± 12.9 years (range: 19–73 years), of which 52 were male and 15 were female. The average body mass index (BMI) was 24.1 ± 3.3 kg/m^2^ (range: 16.9–32.5 kg/m^2^). The mean HHS in the left hip for all patients was 75.6 ± 16.6, and the mean HHS of the right hip was 75.0 ± 18.0. ONFH was idiopathic in 29 patients, secondary to steroid use in 16 patients, and associated with alcohol use in 22 patients. According to the ARCO stage, 16 hips had stage II disease, 56 hips had stage III disease, and 30 hips had stage IV disease. CJFH types were as follows: L1, 19 hips; L2, 14 hips; L3, 67 hips; M, 0 hips; and C, 2 hips.

### 2.2. Staging and Typing

**Figure 2:**
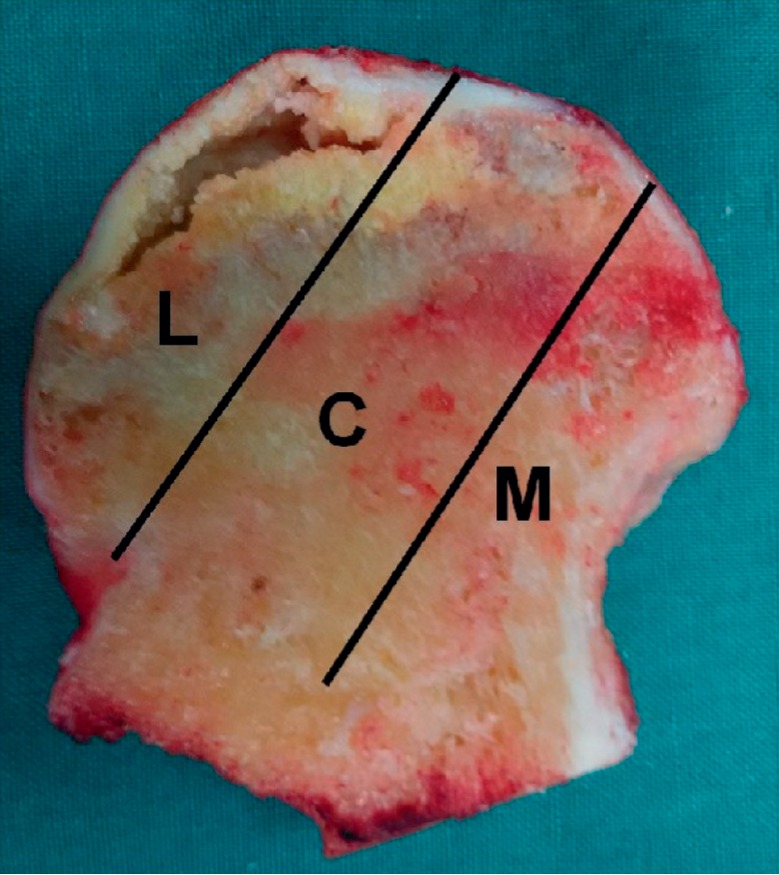
Image of coronal section of the femoral head showing three pillars of the femoral head: lateral (30%), central (40%), and medial (30%).

The stages by ARCO classification system were stage II in 16 hips, stage III in 56 hips, and stage IV in 30 hips. All subjects with ONFH underwent an MRI or CT evaluation according to CJFH type [19] (Figure 1) based on three pillars (Figure 2). According to the involvement of necrosis in the three pillars on a mid-coronal section on MRI or CT, ONFH location was divided into three types (M, C, and L), and the intact degree of the lateral pillar was divided into subtypes (L1, L2, and L3). Using this type to predict the prognosis of the patients with ONFH and the efficacy of joint-preserving surgery for ONFH, the CJFH types were type L1 in 19 hips, type L2 in 14 hips, type L3 in 67 hips, type M in 0 hips, and type C in 2 hips.

### 2.3. Measurements of T and B Lymphocyte Subsets

Peripheral vein blood (2 ml) was also collected in sterile anticoagulation tubes from the included patients and the healthy controls on the day of admission, and the samples were centrifuged at 1500 rpm for 10 min at 24°C, after which the white blood cells (WBCs) were collected with a sterile Pasteur pipette and placed on the surface of a Hypaque Ficoll and centrifuged at 500 rpm for 30 min at 4°C. The lymphocytes were obtained from the interface between the Ficoll and plasma, after which the cell suspensions were washed three times and resuspended in a Ca- and Mg-free phosphate buffered saline. The count, percentage, and ratio of viable T lymphocyte and B lymphocyte subsets, including CD4-positive T cells, CD8-positive T cells, CD5^+^CD19^+^ B-1cells, and CD5^−^CD19^+^ B-2 cells, were measured by flow cytometry (CellQuest program; Becton Dickinson, Franklin Lakes, NJ, USA), and the CD4 : CD8 ratio was calculated. The T lymphocyte and B lymphocyte subsets were measured by two experienced independent investigators using the same instrumentation who were unaware of the study design in order to enhance measurement accuracy. All samples were duplicated during measurements. The two investigators had the same professional qualifications and were trained before the initial measurement. The data extraction and quality assessment was independently performed by two of the authors (B. L. W. and W. S.). If there were any disagreements, all of the authors discussed them until consensus can be reached.

### 2.4. Statistical Analysis

The data were analyzed using SPSS version 21.0 statistical software (SPSS Inc., Chicago, IL, USA). Quantitative variables are reported as mean ± standard deviation (SD). Nonpaired *t*-tests were used to compare the count, percentage, and ratio of T and B lymphocyte subsets between different groups. One-way analysis of variance was used to compare the count, percentage, and ratio of T and B lymphocyte subsets among different stages, types, and etiology groups. For statistically significant differences, groups were compared using the least significant difference (LSD) test. Pearson's correlation test was used to identify the correlation between the T and B lymphocyte subsets and length of disease history. All tests were two-tailed at the 5% level of significance.

## 3. Results

Demographic data were comparable between the two groups (Table 2). The total lymphocyte count, CD3^+^T cells, Ts cells (CD3^+^CD8^+^), B-1 cell count, and B-1 cells (CD5^+^CD19^+^) in peripheral blood were significantly higher in the patients with ONFH than those in the control subjects (*P* < 0.05) (Table 3), but there are no significant differences between the ONFH group and the control group in the percentage of lymphocytes, total T lymphocytes, Th cells and Ts cells, Th cells (CD3^+^CD4^+^), Th/Ts, B-2 cell count, and B-2 cells (CD5^−^CD19^+^) (*P* > 0.05). In terms of the different ONFH etiologies (steroid use, excessive alcohol intake, or idiopathic origin), the total lymphocyte count and Ts cells (CD3^+^CD8^+^) in peripheral blood were significantly lower in the ONFH patients induced by excessive alcohol intake (1890.9 ± 598.8 cell/*μ*l and 464.0 ± 185.2 cell/*μ*l) than those in the idiopathic ONFH patients (2309.7 ± 711.5 cell/*μ*l and 630.7 ± 305.7 cell/*μ*l) (*P*=0.035 and *P*=0.023).

Significant differences are observed in the Ts cells (CD3^+^CD8^+^), Th/Ts, the percentage of T lymphocytes, and Ts cells (CD3^+^CD8^+^) among different ARCO stages in the ONFH patients (*P*=0.016, *P*=0.011, *P* < 0.001, and *P* < 0.001) (Table 4). The Ts cells (CD3^+^CD8^+^) in the patients with ARCO IV stage were significantly smaller than those in the ONFH patients with ARCO III stage (*P*=0.004). The percentage of T lymphocytes and Ts cells (CD3^+^CD8^+^) in the patients with ARCO IV stage was significantly smaller than that in the ONFH patients with ARCO II and III stages (*P* < 0.001). The Th/Ts in the patients with ARCO IV stage was significantly larger than that in the ONFH patients with ARCO II and III stages (*P*=0.017 and *P*=0.006).

Significant differences are observed in the Ts cells (CD3^+^CD8^+^), Th/Ts, and the percentage of Th cells (CD3^+^CD4^+^) and Ts cells (CD3^+^CD8^+^) among different CJFH types in the ONFH patients (*P*=0.001, *P*=0.001, *P*=0.038, and *P* < 0.001) (Table 4). The Ts cells (CD3^+^CD8^+^) in the patients with L1 type were significantly larger than those in the ONFH patients with L3 type and L2 type (*P* < 0.001 and *P*=0.012). The Th/Ts in the patients with L1 type was significantly lower than that in the ONFH patients with L3 (*P* < 0.001). The percentage of Ts cells (CD3^+^CD8^+^) in the patients with L3 type was significantly smaller than that in the ONFH patients with L1 type and L2 type (*P* < 0.001 and *P*=0.002). The percentage of Ts cells (CD3^+^CD4^+^) in the patients with L1 type was significantly smaller than that in the ONFH patients with L2 type and L3 type (*P*=0.031 and *P*=0.005).

The results of Pearson's correlation test between the count, percentage, and ratio of T and B lymphocyte subsets in peripheral blood and length of disease history for the ONFH patients showed that the Ts cells (CD3^+^CD8^+^), Th/Ts, and the percentage of T lymphocytes and Ts cells (CD3^+^CD8^+^) significantly correlated with the length of disease history (25.0 ± 53.0 months) (*P*=0.021, *P*=0.001, *P*=0.029, and *P*=0.001).

## 4. Discussion

In this study, our findings showed that the total lymphocyte count, CD3^+^T cells, Ts cells (CD3^+^CD8^+^), B-1 cell count, and B-1 cells (CD5^+^CD19^+^) were significantly higher in the patients with ONFH than those in the control subjects. The percentage of inhibitory T lymphocytes in the patients with ARCO IV stage was significantly smaller than that in the ONFH patients with ARCO II and III stages. The percentage of inhibitory T lymphocytes in patients with CJFH type L3 was significantly smaller than that in the patients with types L1 and L2. The total lymphocyte count and Ts cells (CD3^+^CD8^+^) were significantly lower in the ONFH patients induced by excessive alcohol intake than those in the idiopathic ONFH patients.

The essence of the pathological process of avascular necrosis of femoral head is that the destruction of bone is greater than the generation of bone. Femoral head collapse is actually stronger in osteolysis than osteogenesis. Osteoblasts and osteoclasts are two main types of cells in the process of bone reconstruction, which regulate the generation, activity, and interaction of OB and OC, and can coordinate the dynamic balance of bone formation and bone resorption. In the process of bone reconstruction, the functional activities of OB and OC do not exist in isolation, but there is an interaction between the two cells. Many experiments of OB and OC coculture have proved that if the coupling of the two is out of balance, it will lead to bone metabolism disorder [21, 22]. We mentioned earlier that T cells regulate the balance between osteoblasts and osteoclasts by secreting OPG or expressing RANKL on the cell surface [14].

In our study, the percentage of inhibitory T lymphocytes in patients with ARCO IV/CJFH type L3 was significantly smaller than that in the patients with ARCO II and III/CJFH types L1 and L2. Inhibitory T cells can bind to B7-1 and B7-2 in osteoclast precursors via cytotoxic T lymphocyte associated protein (CTLA-4), and the combination inhibits osteoclast damage to bone [15]. Meanwhile, regulatory T cells also produce IL-4, IL-10, TGF-*β*, etc. These cytokines not only have anti-inflammatory effects but also play an important role in inhibiting the occurrence of osteoclasts [23, 24]. These results suggest that the decrease in the number of inhibitory T cells may be related to the progression of ONFH. ARCO IV/CJFH L3 patients usually present with marked bone destruction, femoral head collapse, and joint space narrowing.

In our study, we found that B1 cells in the peripheral blood of ONFH patients were significantly higher than those in the non-ONFH group. However, there was no significant difference between patients with ONFH caused by different etiologies. B1 cells play an important role in the immune system. B1 cells are mainly involved in the innate immune response and can recognize a variety of bacterial surface antigens and some autoantigens and produce antibodies [25]. It has been found that the natural antibodies produced by it also play an important role in removing apoptotic cells and forming granulomas [26, 27]. Previous studies have shown that peripheral blood activation of B cells is more frequent in patients with nontraumatic femoral head necrosis, especially in patients with glucocorticosteroid-related and alcohol-related ONFH [28]. This may be related to the progression of ONFH. As a subset of B cells, B1 cells may be involved in this process.

In addition, we found that in the ONFH patient group, the total lymphocyte count and Ts cells (CD3^+^CD8^+^) in the peripheral blood of patients with ONFH caused by excessive drinking were lower than those of patients with idiopathic ONFH. Studies have shown that alcohol can have some negative effects on the immune system [29], which may play a role in the development of alcoholic ONFH, but the specific mechanism is not fully supported by basic experiments, so we will not discuss too much here.

This study has some limitations. Firstly, the sample size was relatively small and the analysis was not stratified by age or gender. Secondly, our observation was limited to inpatients without long-term follow-up. If this study is based on larger and longer clinical observations, lymphocyte and subgroup levels can more accurately reflect the progression of osteonecrosis due to different causes. Thirdly, the control group was matched only for age, BMI, and other factors, but not for other factors, such as alcohol intake. Fourthly, our study is conducted in a single center, and if a large-scale multicenter study is carried out, there may be more valuable findings. Finally, more basic and clinical studies are needed for the clinical guidance of the results.

In conclusion, our study found that immunomodulatory cells are closely related to the pathogenesis of ONFH, especially inhibitory T lymphocytes, which play an important regulatory role in the bone mass balance of the femoral head after the occurrence of ischemic necrosis of the femoral head. Inhibitory T cells decreased in peripheral blood of patients with end-stage femoral head necrosis (CJFH L3/ARCO IV), which may be closely related. In addition, the elevated level of B1 cells in the peripheral blood of ONFH patients may also be of great significance, which has not been reported yet and needs to be further explored. Finally, due to the limitation of sample size, we have not found any differences in other T and B cell subsets between ONFH patients and non-ONFH patients.

## Figures and Tables

**Table 1:** The demographics of patients with ONFH.

Demographic	No./mean (SD)
Patients (M/F)	67
Unilateral	32
Bilateral	35
Hips	102
Male	52
Female	15
Mean age (year)	43.6 (12.9)
Mean BMI (kg/m^2^)	24.1 (3.3)
Mean HHS (score)	
Left	75.6 (16.6)
Right	75.0 (18.0)
Mean length of disease history (month)	25.0 (53.0)
Etiology	
Idiopathic	29
Corticosteroids	16
Alcohol	22
ARCO stage, hips	
Stage II	16
Stage III	56
Stage IV	30
CJFH classification, hips	
L1	19
L2	14
L3	67
M	0
C	2

**Table 2:** Demographic data of the ONFH group and the control group.

	ONFH group (*n* = 67)	Control group (*n* = 58)	*P* value
Age (years)	43.6 ± 12.9	47.3 ± 9.3	0.067
Gender (male/female)	52/15	43/15	0.650
Height (cm)	170.7 ± 7.5	169.2 ± 5.6	0.204
Weight (kg)	70.3 ± 10.8	68.8 ± 13.9	0.523
BMI (kg/m^2^)	24.1 ± 3.3	25.4 ± 4.3	0.070

**Table 3:** Comparison of T and B lymphocyte subsets between the ONFH group and the control group.

	ONFH group	Control group	*P* value
Total lymphocyte count (cell/*μ*l)	2071.6 ± 708.2	1777.8 ± 690.2	0.021
CD3^+^T cell (cell/*μ*l)	1495.7 ± 552.0	1294.9 ± 517.5	0.038
Ts cell (CD3^+^CD8^+^) (cell/*μ*l)	549.0 ± 260.0	447.4 ± 217.0	0.020
B-1 cell count (cell/*μ*l)	102.0 ± 93.7	34.4 ± 29.9	<0.001
B-1 cell (CD5^+^CD19^+^) (%)	4.9 ± 4.0	1.9 ± 1.4	<0.001

**Table 4:** Comparison of T and B lymphocyte subsets among different ARCO stages and CJFH types in the ONFH group.

	ARCO II stage	ARCO III stage	ARCO IV stage	*P* value
Ts cells (CD3^+^CD8^+^) (cells/*μ*l)	530.4 ± 197.2	589.1 ± 275.1	423.5 ± 218.2	0.016
Th/Ts	1.6 ± 0.9	1.7 ± 0.7	2.2 ± 1.0	0.011
The percentage of T lymphocytes (%)	76.1 ± 7.1	74.4 ± 7.2	63.6 ± 10.4	<0.001
The percentage of Ts cells (CD3^+^CD8^+^) (%)	29.5 ± 8.1	27.7 ± 7.6	20.3 ± 5.9	<0.001

	CJFH L1 type	CJFH L2 type	CJFH L3 type	
Ts cells (CD3^+^CD8^+^) (cells/*μ*l)	743.3 ± 317.7	528.2 ± 189.7	473.8 ± 221.4	0.001
Th/Ts	1.2 ± 0.4	1.7 ± 0.9	2.1 ± 0.8	0.001
The percentage of Ts cells (CD3^+^CD8^+^) (%)	31.7 ± 5.7	30.0 ± 9.6	23.1 ± 6.7	<0.001
The percentage of Th cells (CD3^+^CD4^+^) (%)	36.7 ± 7.1	43.5 ± 9.0	43.3 ± 9.0	0.038

## Data Availability

All data generated and analyzed during the study are available from the corresponding author upon request.

## Supplementary Materials

Supplementary Materials The provided supplementary file is the data of T lymphocyte and B lymphocyte subsets in the included patients.
