# Supplementary material for: The Role of Immune Regulatory Cells in Nontraumatic Osteonecrosis of the Femoral Head: A Retrospective Clinical Study
Source: Biomed Res Int. 2019 Nov 20;2019:1302015. doi: 10.1155/2019/1302015 (PMC6886356; doi:10.1155/2019/1302015)
Supplement: Supplementary Materials — The provided supplementary file is the data of T lymphocyte and B lymphocyte subsets in the included patients. [file 1302015.f1.pdf]

病案号	组别	性别	年龄	身高	体重	单双侧	病因	
1,400,184	1	2	31	154	57	1	1	
1,405,597	1	2	31	165	50	2	1	
1,365,591	1	1	60	180	75	2	1	
1,406,756	1	1	49	170	60	1	1	
1,406,876	1	1	73	160	55	2	3	
1,408,117	1	1	57	176	80	2	2	
1,408,687	1	1	54	174	75	1	3	
1,409,299	1	1	38	178	71	2	3	
1,411,569	1	2	58	168	55	2	3	
1,411,994	1	1	57	170	75	1	3	
1,412,719	1	1	42	170	84	1	2	
1,412,894	1	1	33	175	64	2	3	
1,413,238	1	1	34	170	82	1	3	
1,264,404	1	1	60	163	73	2	1	
1,414,450	1	1	43	160	65	2	3	
1,416,033	1	2	63	158	52	1	2	
1,419,586	1	2	44	167	85	1	1	
1,429,922	1	1	42	170	75	2	1	
1,425,234	1	1	22	180	60	2	3	
1,437,858	1	1	27	173	77	1	3	
1,437,760	1	1	50	165	56	1	2	
1,440,509	1	1	56	163	60	1	2	
1,440,466	1	1	26	181	74	1	3	
1,440,484	1	1	29	180	68	1	3	
1,440,703	1	1	46	166	66	1	2	
1,441,812	1	1	34	181	76	1	2	
1,442,722	1	1	53	170	75	2	2	
1,353,751	1	2	20	167	54	2	1	
1,448,265	1	1	51	168	75	1	2	
1,448,602	1	1	26	170	80	2	1	
1,449,749	1	1	51	180	82	1	2	
1,450,493	1	1	22	180	72	2	1	
1,450,305	1	1	46	169	74	2	2	
1,450,963	1	2	37	158	73	1	3	
1,451,209	1	2	46	158	70	1	3	
1,451,371	1	1	34	171	85	2	3	
1,451,557	1	1	45	170	75	2	2	
1,452,319	1	1	51	178	95	1	2	
1,452,591	1	1	30	175	85	2	2	
1,283,183	1	2	61	158	58	2	3	
1,453,984	1	1	32	185	84	1	3	
1,453,674	1	1	32	185	71	2	1	
1,454,115	1	1	62	167	71	1	3	
1,455,032	1	1	39	176	80	1	3	
1,456,077	1	2	51	163	69	1	3	
1,456,214	1	1	45	170	66	1	2	
1,456,804	1	1	32	177	80	2	2	
1,429,275	1	1	26	183	69	2	3	
1,456,887	1	1	43	172	96	2	3	
1,456,912	1	1	33	170	86	1	3	
1,457,556	1	1	54	178	70	2	3	
1,458,267	1	1	47	179	68	1	1	
1,460,963	1	1	26	172	72	2	1	
1,460,723	1	1	53	170	68	2	3	
1,459,737	1	1	31	174	86	1	3	

淋巴细胞总数	淋巴细胞百分数	淋巴细胞计数	T淋巴细胞CD3	T辅助诱导CD3CD	T抑制杀伤CD3CD
2.130	27.1	1,840	1,426	773	626
1.060	18.9	1,060	815	515	289
0.800	14.2	800	505	282	214
3.310	44.6	3,310	2,012	861	1,102
1.450	16.4	1,450	712	509	175
0.670	7.9	670	312	204	104
1.270	12.5	1,270	758	415	304
4.170	33.4	4,170	3,178	1,314	1,389
2.210	40.1	2,210	1,313	800	442
2.730	28.6	2,730	1,646	816	713
2.750	33.3	2,750	1,372	941	404
1.550	30.3	1,550	1,203	527	575
1.180	21.3	1,030	795	478	289
1.980	22.2	1,980	1,529	1,154	299
2.660	16.3	2,660	1,519	902	553
1.070	24.2	1,070	782	489	305
1.780	23.6	1,780	1,451	892	504
1.710	28.6	1,710	1,264	947	284
2.950	38.6	2,950	1,587	723	811
2.940	43.6	2,940	2,475	1,182	1,114
2.250	28.9	2,550	2,055	1,288	755
1.300	26.3	1,210	963	571	364
2.320	45.9	2,320	1,865	1,199	626
2.770	40.1	2,120	1,259	681	547
2.030	31.2	2,030	1,328	834	414
1.930	28.0	1,930	1,372	758	550
1.910	26.5	1,910	1,308	970	296
2.030	29.9	2,030	1,358	696	617
1.990	35.1	1,990	1,473	846	593
1.500	33.0	1,500	1,238	477	701
1.530	23.9	1,530	1,258	644	546
1.990	36.5	1,990	1,457	890	507
2.000	32.0	2,000	1,172	710	366
2.220	36.8	2,220	1,685	722	921
1.920	38.9	1,920	1,321	739	401
1.550	20.8	1,770	1,333	784	490
2.940	40.9	2,940	2,246	1,367	676
2.030	28.7	2,030	1,520	997	499
2.960	36.1	2,960	2,072	1,107	924
2.520	42.1	2,520	1,462	1,036	396
4.280	46.5	4,280	3,548	2,204	1,395
1.590	27.4	1,590	949	418	452
2.560	30.5	2,590	1,987	1,038	831
2.030	21.6	2,030	1,701	1,009	615
1.800	34.6	1,800	1,413	819	553
1.550	24.8	1,550	1,231	859	341
1.930	35.0	1,930	1,357	687	591
2.370	35.2	2,530	1,822	898	873
1.710	27.6	1,710	1,206	814	373
2.200	18.9	2,200	1,698	823	814
2.430	39.5	2,430	1,910	1,560	326
2.080	38.4	2,080	1,649	1,148	422
3.360	36.2	3,360	2,644	1,788	759
2.110	26.9	2,110	1,317	844	414
2.750	32.2	2,750	1,991	1,139	789

T总百分比	T辅助百分比	T抑制百分比	Th比Ts	B1绝对值	B2绝对值	B细胞亚群B1CD5CD1
77.5	42.0	34.0	1.24	82	223	4.45
76.9	48.6	27.3	1.78	13	42	1.20
63.1	35.3	26.7	1.32	25	72	3.07
60.8	26.0	33.3	0.78	66	203	2.00
49.1	35.1	12.1	2.90	238	216	16.40
46.6	30.4	15.5	1.96	56	109	8.33
59.7	32.7	23.9	1.37	71	124	5.60
76.2	31.5	33.3	0.95	195	331	4.67
59.4	36.2	20.0	1.81	32	108	1.47
60.3	29.9	26.1	1.15	188	379	6.88
49.9	34.2	14.7	2.33	310	371	11.28
77.6	34.0	37.1	0.92	91	187	5.87
77.2	46.4	28.1	1.65	76	50	7.40
77.2	58.3	15.1	3.86	108	219	5.47
57.1	33.9	20.8	1.63	13	130	0.47
73.1	45.7	28.5	1.60	16	69	1.47
81.5	50.1	28.3	1.77	13	20	0.73
73.9	55.4	16.6	3.34	94	239	5.50
53.8	24.5	27.5	0.89	547	547	18.55
84.2	40.2	37.9	1.06	59	276	2.00
80.6	50.5	29.6	1.71	56	2,477	2.20
79.6	47.2	30.1	1.57	58	65	4.80
80.4	51.7	27.0	1.91	122	146	5.25
59.4	32.1	25.8	1.24	93	139	4.40
65.4	41.4	20.4	2.01	86	202	4.25
71.1	39.3	28.5	1.38	61	199	3.15
68.5	50.8	15.5	3.28	146	269	7.65
66.9	34.3	30.4	1.13	177	166	8.70
74.0	42.5	29.8	1.43	122	126	6.15
82.5	31.8	46.7	0.68	100	107	6.65
82.2	42.1	35.7	1.18	60	89	3.95
73.2	44.7	25.5	1.75	82	146	4.10
58.6	35.5	18.3	1.94	234	239	11.70
75.9	32.5	41.5	0.78	120	112	5.40
68.8	38.5	20.9	1.84	186	164	9.70
75.3	44.3	27.7	1.60	23	171	1.30
76.4	46.5	23.0	2.02	176	243	6.00
74.9	49.1	24.6	2.00	54	254	2.65
70.0	37.4	31.2	1.20	284	274	9.58
58.0	41.1	15.7	2.62	97	377	3.84
82.9	51.5	32.6	1.58	103	347	2.40
59.7	26.3	28.4	0.93	291	311	18.33
76.7	41.8	32.1	1.30	73	227	2.81
83.8	49.7	30.3	1.64	18	157	0.90
78.5	45.5	30.7	1.48	47	146	2.60
79.4	55.4	22.0	2.52	40	131	2.58
70.3	35.6	30.6	1.16	212	235	11.00
72.0	35.5	34.5	1.03	128	233	5.05
70.5	47.6	21.8	2.18	13	61	0.75
77.2	37.4	37.0	1.01	107	224	4.85
78.6	64.2	13.4	4.79	119	377	4.90
79.3	55.2	20.3	2.72	19	150	0.89
78.7	53.2	22.6	2.35	180	323	5.35
62.4	40.0	19.6	2.04	42	302	2.00
72.4	41.4	28.7	1.44	206	385	7.50

B细胞亚群B2CD5CD19
12.10
3.83
9.00
6.13
14.87
16.20
9.73
7.93
4.87
13.88
13.49
12.07
4.84
11.07
4.87
6.47
1.13
14.00
18.55
9.40
9.70
5.35
6.30
6.55
9.95
10.30
14.10
8.20
6.35
7.15
5.80
7.35
11.95
5.05
8.55
9.67
8.25
12.50
9.24
14.95
8.10
19.53
8.75
7.75
8.10
8.43
12.18
9.20
3.55
10.20
15.50
7.19
960.00
14.30
14.00

1,460,311	1	2	55	165	62	1	3
1,461,397	1	1	61	170	65	2	2
1,465,651	1	1	48	184	70	2	2
1,466,283	1	2	61	158	59	1	2
1,468,602	1	1	38	175	73	2	2
1,468,323	1	1	49	170	65	1	2
1,469,414	1	1	50	174	70	1	2
1,470,693	1	1	30	165	60	2	1
1,472,361	1	1	56	170	69	2	1
1,473,586	1	2	60	163	60	2	3
1,487,573	1	1	19	172	50	2	1
1,489,611	1	1	55	160	46	2	3
54,145	2	1	47	162	54	#NULL!	#NULL!
1,233,478	2	1	52	165	60	#NULL!	#NULL!
98,235	2	2	52	166	63	#NULL!	#NULL!
1,105,194	2	1	52	170	81	#NULL!	#NULL!
1,290,918	2	2	54	165	65	#NULL!	#NULL!
1,330,113	2	1	51	180	95	#NULL!	#NULL!
1,405,675	2	1	42	170	67	#NULL!	#NULL!
1,405,905	2	1	56	170	81	#NULL!	#NULL!
1,426,793	2	1	52	173	84	#NULL!	#NULL!
1,439,770	2	1	31	178	80	#NULL!	#NULL!
1,456,764	2	1	48	178	90	#NULL!	#NULL!
1,480,440	2	1	55	166	63	#NULL!	#NULL!
2,202,044	2	1	56	178	90	#NULL!	#NULL!
2,252,301	2	1	57	167	60	#NULL!	#NULL!
1,439,770	2	2	31	178	80	#NULL!	#NULL!
1,456,764	2	1	48	178	90	#NULL!	#NULL!
2,257,930	2	1	50	165	69	#NULL!	#NULL!
2,259,363	2	2	51	173	65	#NULL!	#NULL!
2,259,432	2	1	40	166	55	#NULL!	#NULL!
2,259,453	2	1	57	165	71	#NULL!	#NULL!
2,259,465	2	1	50	166	68	#NULL!	#NULL!
2,259,551	2	1	58	166	75	#NULL!	#NULL!
2,259,865	2	1	55	169	45	#NULL!	#NULL!
2,259,891	2	2	56	164	65	#NULL!	#NULL!
2,259,997	2	2	49	164	69	#NULL!	#NULL!
2,260,000	2	1	39	178	91	#NULL!	#NULL!
2,260,582	2	1	66	168	55	#NULL!	#NULL!
2,260,976	2	1	50	170	58	#NULL!	#NULL!
2,261,006	2	1	51	160	45	#NULL!	#NULL!
2,261,054	2	2	28	169	54	#NULL!	#NULL!
2,261,059	2	1	40	178	84	#NULL!	#NULL!
2,261,070	2	1	58	164	70	#NULL!	#NULL!
2,261,078	2	1	49	165	70	#NULL!	#NULL!
2,261,145	2	2	52	166	51	#NULL!	#NULL!
2,261,876	2	1	44	170	63	#NULL!	#NULL!
262,339	2	2	50	165	60	#NULL!	#NULL!
2,263,164	2	1	41	169	98	#NULL!	#NULL!
2,263,571	2	1	54	171	77	#NULL!	#NULL!
2,264,232	2	2	49	164	60	#NULL!	#NULL!
2,264,267	2	1	45	169	59	#NULL!	#NULL!
2,264,379	2	1	50	173	70	#NULL!	#NULL!
2,264,475	2	1	54	168	64	#NULL!	#NULL!
2,264,691	2	1	25	170	49	#NULL!	#NULL!
2,264,993	2	1	47	164	75	#NULL!	#NULL!

2.060	22.2	2,060	1,685	896	731
2.150	30.5	2,150	1,662	1,097	544
1.800	22.6	1,800	1,447	731	380
1.270	27.4	1,270	972	682	278
1.250	26.5	1,250	911	645	241
2.400	34.1	2,400	1,927	1,346	478
1.680	28.0	1,680	1,369	785	558
1.490	30.2	1,490	1,220	472	626
2.830	45.6	2,830	1,973	1,412	538
2.300	50.2	2,300	1,695	1,168	324
0.690	11.0	870	781	364	345
2.360	27.0	2,360	1,749	918	505
1.450	33.6	1,450	1,146	500	619
0.690	6.4	690	503	228	257
1.640	19.5	1,640	1,381	1,012	351
2.330	38.8	2,330	1,312	354	841
1.410	30.3	1,410	943	471	385
1.470	33.1	1,470	957	664	226
2.220	34.1	2,470	2,216	855	1,178
1.570	33.6	1,460	1,184	707	448
1.770	26.9	1,770	1,292	812	446
1.980	29.8	1,980	1,528	998	489
1.540	29.4	1,540	996	582	360
0.790	21.5	790	577	382	159
1.650	29.2	2,850	1,870	1,223	604
2.030	53.0	2,030	1,411	745	536
3.010	40.7	1,980	1,582	998	489
1.540	29.4	1,540	996	582	360
1.550	38.5	1,550	1,297	930	327
0.630	6.6	630	489	225	247
1.400	24.0	1,900	1,465	847	534
1.980	33.9	1,980	1,232	840	388
1.940	34.4	1,940	1,141	755	363
2.150	17.5	2,150	1,423	1,077	329
0.720	11.5	720	566	362	186
1.040	13.0	1,040	760	422	326
1.980	38.5	1,980	1,560	895	667
2.360	39.1	2,260	1,774	1,110	524
0.820	14.5	820	560	290	267
2.000	27.8	2,890	2,043	1,416	590
0.880	8.5	880	649	414	238
3.330	39.2	3,790	2,880	1,857	868
2.930	35.4	2,420	1,822	1,280	508
2.070	36.8	2,920	1,784	1,215	610
1.010	31.7	1,010	829	521	293
1.280	35.2	1,280	922	722	196
2.100	28.1	2,100	1,457	714	624
1.420	30.0	1,470	1,045	692	337
1.610	35.5	1,610	1,278	845	407
2.040	30.5	1,770	1,253	878	350
1.420	22.7	1,420	821	508	273
1.330	23.4	1,330	1,021	584	408
2.410	38.7	2,410	1,848	1,294	525
1.310	18.4	1,310	983	634	351
2.560	45.0	2,790	2,126	1,283	709
1.540	26.0	1,540	1,153	671	434

80.5	43.5	35.5	1.23	8	155	0.38
77.3	51.0	25.3	2.02	73	280	3.38
80.4	40.6	21.1	1.92	35	103	1.94
76.5	53.7	21.9	2.45	20	115	1.60
72.9	51.6	19.3	2.67	75	98	6.00
80.3	56.1	19.9	2.82	44	167	1.85
81.5	46.7	33.2	1.41	23	215	1.35
81.9	31.7	42.0	0.75	31	86	2.10
69.7	49.9	19.0	2.63	0	20	0.00
73.7	50.8	14.1	3.60	74	251	3.22
89.8	41.8	39.7	1.05	14	45	1.65
74.1	38.9	21.4	1.82	211	214	8.92
79.0	34.5	42.7	0.81	32	171	2.20
72.9	33.0	37.3	0.88	16	92	2.27
84.2	61.7	21.4	2.88	14	109	0.87
56.3	15.2	36.1	0.42	8	67	0.33
66.9	33.4	27.3	1.22	80	179	5.67
65.1	45.2	15.4	2.94	78	216	5.33
89.7	34.6	47.7	0.73	15	142	0.60
81.1	48.4	30.7	1.58	7	138	0.47
73.0	45.9	25.2	1.82	33	196	1.87
79.9	50.4	24.7	2.04	33	243	1.67
64.7	37.8	23.4	1.62	51	165	3.33
73.0	48.4	20.1	2.41	23	108	2.93
65.6	42.9	21.2	2.02	91	279	3.20
69.5	36.7	26.4	1.39	41	311	2.00
79.9	50.4	24.7	2.04	33	243	1.67
64.7	37.8	23.4	1.62	51	165	3.33
83.7	60.0	21.1	2.84	5	168	0.33
77.6	35.7	39.2	0.91	0	26	0.07
77.1	44.6	28.1	1.59	67	289	3.53
62.2	42.4	19.6	2.16	82	330	4.13
58.8	38.9	18.7	2.08	40	217	2.07
66.2	50.1	15.3	3.27	54	314	2.53
78.6	50.3	25.9	1.94	0	22	0.00
73.1	40.6	31.3	1.30	0	35	0.00
78.8	45.2	33.7	1.34	86	263	4.33
78.5	49.1	23.2	2.12	62	345	2.73
68.3	35.4	32.5	1.09	26	119	3.20
70.7	49.0	20.4	2.40	83	410	2.87
73.8	47.0	27.1	1.73	13	117	1.47
76.0	49.0	22.9	2.14	99	783	2.60
75.3	52.9	21.0	2.52	26	294	1.07
61.1	41.6	20.9	1.99	148	685	5.07
82.1	51.6	29.0	1.78	9	53	0.93
72.0	56.4	15.3	3.69	3	90	0.20
69.4	34.0	29.7	1.14	11	181	0.53
71.1	47.1	22.9	2.06	31	137	2.13
79.4	52.5	25.3	2.08	10	164	0.60
70.8	49.6	19.8	2.51	48	257	2.73
57.8	35.8	19.2	1.86	7	75	0.47
76.8	43.9	30.7	1.43	24	169	1.80
76.7	53.7	21.8	2.46	39	236	1.60
75.0	48.4	26.8	1.81	24	219	1.87
76.2	46.0	25.4	1.81	19	299	0.67
74.9	43.6	28.2	1.55	29	203	1.87

7.50
13.00
5.74
9.07
7.85
6.95
12.80
5.75
0.70
10.91
5.15
9.06
11.18
13.33
6.67
2.87
12.73
14.67
5.73
9.47
11.07
12.27
10.73
13.67
9.80
15.33
12.27
10.73
10.87
4.07
15.20
16.67
11.20
14.60
3.00
3.33
13.27
15.27
14.47
14.20
13.33
20.67
12.13
23.47
5.20
7.07
8.60
9.33
10.20
14.53
5.27
12.73
9.80
16.73
10.73
13.20

2,265,098	2	2	52	150	70	#NULL!	#NULL!
2,265,099	2	1	49	173	92	#NULL!	#NULL!
2,265,100	2	1	42	166	75	#NULL!	#NULL!
2,265,287	2	2	55	169	70	#NULL!	#NULL!
2,265,438	2	1	46	167	70	#NULL!	#NULL!
2,265,781	2	2	38	170	100	#NULL!	#NULL!
2,265,916	2	2	24	172	56	#NULL!	#NULL!
2,266,107	2	1	51	177	55	#NULL!	#NULL!
2,267,535	2	1	50	170	40	#NULL!	#NULL!
1,226,004	2	1	49	172	68	#NULL!	#NULL!
2,267,783	2	1	43	175	67	#NULL!	#NULL!
1,220,933	2	1	42	167	49	#NULL!	#NULL!
2,266,511	2	2	42	172	68	#NULL!	#NULL!
2,227,975	2	1	16	171	75	#NULL!	#NULL!

2.320	34.7	2,320	1,749	1,283	432
1.730	29.0	1,730	1,337	787	519
1.620	26.1	1,620	1,275	773	428
1.790	31.0	1,790	1,190	967	140
2.260	32.6	2,260	1,559	644	841
1.530	23.3	1,850	1,393	744	568
1.680	32.8	1,680	1,384	771	415
2.300	38.0	2,300	1,854	1,281	543
0.680	8.7	680	574	394	184
1.270	15.2	1,270	819	654	141
3.520	34.7	3,250	2,267	1,461	729
1.450	17.9	1,450	916	666	236
1.890	36.4	700	454	284	155
2.900	30.9	2,900	2,256	1,035	992

75.4	55.3	18.6	2.97	32	311	1.40
77.3	45.5	30.0	1.52	25	206	1.47
78.7	47.7	26.4	1.81	52	196	3.20
66.5	54.0	7.8	6.92	24	170	1.33
69.0	28.5	37.2	0.77	32	310	1.40
75.3	40.2	30.7	1.31	51	231	2.73
82.4	45.9	24.7	1.86	13	133	0.80
80.6	55.7	23.6	2.36	21	219	0.93
84.4	57.9	27.1	2.14	0	24	0.07
64.5	51.5	11.1	4.64	16	169	1.27
64.4	41.5	20.7	2.00	17	228	0.47
63.2	45.9	16.3	2.82	5	184	0.33
64.8	40.6	22.1	1.84	11	87	1.63
77.8	35.7	34.2	1.04	43	410	1.47

13.40
11.93
12.07
9.47
13.73
12.47
7.93
9.53
3.47
13.33
6.47
12.67
12.44
14.13
